# 

**Published:** 1894-08-11

**Authors:** 


PRICE TWOPENCE.
No. 411, Vol. XVI.—Aug. 11th, 1894.
[Registered at the General Post Office as a Newspaper for transmission in the United Kingdom and Abroad.]

WITH EXTRA NURSING SUPPLEMENT
Per Annum, 10s. 6d., post free.
Monthly Parts, 6d.; post free, 8d.
Ditto per Annum, 8s. 6d., post free

### THE HOSPITAL

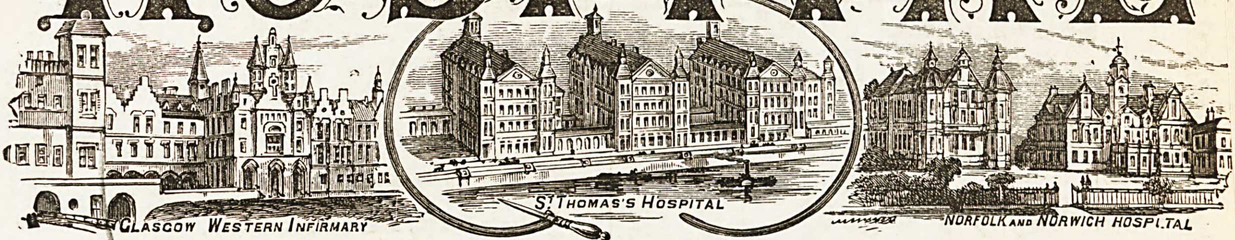

No. 411. Vol. XVI WITH EXTRA NURSING SUPPLEMENT. SATURDAY, AUG. 11TH, 1894.

